# *Burkholderia thailandensis* E264 as a promising safe rhamnolipids’ producer towards a sustainable valorization of grape marcs and olive mill pomace

**DOI:** 10.1007/s00253-021-11292-0

**Published:** 2021-04-20

**Authors:** Alif Chebbi, Massimiliano Tazzari, Cristiana Rizzi, Franco Hernan Gomez Tovar, Sara Villa, Silvia Sbaffoni, Mentore Vaccari, Andrea Franzetti

**Affiliations:** 1 Department of Earth and Environmental Sciences -DISAT, University of Milano-Bicocca, Piazza della Scienza 1 -, 20126 Milano, Italy; 2 Department of Civil, Environmental, Architectural Engineering, and Mathematics, University of Brescia, Via Branze 43, 25123 Brescia, Italy; 3 Sustainability Department, Resource Valorisation Lab, Casaccia Research Center, ENEA, Via Anguillarese 301, 00123 Rome, Italy

**Keywords:** Winery wastes, Olive oil wastes, Circular economy, Rhamnolipids, UPLC-MS, Agricultural wastes

## Abstract

**Abstract:**

Within the circular economy framework, our study aims to assess the rhamnolipid production from winery and olive oil residues as low-cost carbon sources by nonpathogenic strains. After evaluating various agricultural residues from those two sectors, *Burkholderia thailandensis* E264 was found to use the raw soluble fraction of nonfermented (white) grape marcs (NF), as the sole carbon and energy source, and simultaneously, reducing the surface tension to around 35 mN/m. Interestingly, this strain showed a rhamnolipid production up to 1070 mg/L (13.37 mg/g of NF), with a higher purity, on those grape marcs, predominately Rha-Rha C_14_-C_14_, in MSM medium. On olive oil residues, the rhamnolipid yield of using olive mill pomace (OMP) at 2% (w/v) was around 300 mg/L (15 mg/g of OMP) with a similar CMC of 500 mg/L. To the best of our knowledge, our study indicated for the first time that a nonpathogenic bacterium is able to produce long-chain rhamnolipids in MSM medium supplemented with winery residues, as sole carbon and energy source.

**Key points:**

• *Winery and olive oil residues are used for producing long-chain rhamnolipids (RLs).*

• *Both higher RL yields and purity were obtained on nonfermented grape marcs as substrates.*

• *Long-chain RLs revealed stabilities over a wide range of pH, temperatures, and salinities*

**Supplementary Information:**

The online version contains supplementary material available at 10.1007/s00253-021-11292-0.

## Introduction

The European Commission adopted an ambitious Circular Economy Action Plan in 2015 that covers the whole life cycle of a product: from production and consumption to waste management and the secondary materials market (Grimm and Wösten 2018; Kapoor et al. 2020). The concept of circular economy (CE) consists of developing economic models that have minimal effects on the environment, ensuring a reduction in natural resource use and waste generation and promoting waste reuse as valuable co-products (Casson Moreno et al. 2020). The development of a CE concept requires adopting closed-loop systems, facilitating the transformation from a linear economy to a CE to improve economic and environmental sustainability (Dessie et al. 2020; Kaszycki et al. 2020). This model can be especially important in Italy, where agriculture is one of the most productive sectors and, at the same time, responsible for generating large amounts of residues with different proprieties (Vahidzadeh et al. 2021).

In the agricultural sector, the European Union (EU) is the largest producer of wine and olive oil worldwide, accounting for more than 65% and 75% of the global production, respectively (Ferreira et al. 2019). For instance, in Italy, more than 8.6 million tonnes of wine grapes were harvested in 2018 from a production area of 705 thousand hectares (Muhlack et al. 2018). The wine production in 2019 has reached 1.3 million hectoliters in the Lombardy region (northern Italy) (ISTAT 2019). Protected designation of origin (PDO) wines were 781 thousand hectoliters, while protected geographical indication (PGI) production was 535 thousand hectoliters, reaching 36% total production of the historical maximum. For the olive oil sector, the production is mainly concentrated in the south of Italy and exceeded 2.9 million tonnes of olive oil, with 18.9 million tonnes of olive harvested in 2018 (ISTAT 2019). These vital agricultural sectors are associated with diverse adverse effects on the environment, and several life cycle assessment analyses carried out in Italy have pointed out that the generation, management, and treatment of both agricultural residues are the top hot spots to take into consideration for reaching circular supply chains (Guarino et al. 2019). The carbohydrates in plant biomass can be used as a raw material for producing liquid biofuels and valuable biochemicals. However, plant material is heterogeneous and recalcitrant to degradation, even by microorganisms (Singhvi and Gokhale 2019). The use of agricultural residues, including winery and olive sectors, to producing bioactive compounds is generally challenging due to several aspects, such as the heterogeneity of wastes, the toxicity of some other metabolites, the low yields, and the cost of purifications (Dimou et al. 2016; Ochando-Pulido and Martínez-Férez 2017; Ferri et al. 2017).

Chemical surfactants have a huge demand worldwide, and its global market is forecast to reach more than EUR 46 Billion by 2027 (Research 2020). Due to their various toxic effects on the environment, several related sectors are actively working towards replacing them, at least partly, with sustainable biosurfactants (Banat et al. 2010; Li et al. 2020). For that, the global biosurfactant market is growing continuously, with the rhamnolipid market set to witness a gain of over 8% (Singh et al. 2019). The biosurfactants market size has exceeded EUR 1.2 Billion in 2019 and is estimated to grow at over 5.5% compound annual growth rate (CAGR) between 2020 and 2026 (Gminsights 2020). Along with sophorolipids, rhamnolipids (RLs) are considered good candidates for industrial production and applications in numerous fields, e.g., bioremediation of contaminated sites. These glycolipids are among the most studied biosurfactants that display an amphipathic property, i.e., a hydrophobic and a hydrophilic moiety (Banat et al. 2021). Considering these features, RLs are widely commercialized by several companies in the world, e.g., AGAE Technologies and Natsurfact (Banat et al. 2021). These amphiphilic compounds are potent natural emulsifiers that reduce the surface tension of water from roughly 72 mN/m to an average of 25–30 mN/m (Perfumo et al. 2013; Chebbi et al. 2017a). For that, RLs have broadly confirmed interesting features, e.g., the ability to reduce surface and interfacial tensions of solutions, the formation of microemulsions, and the stability over a wide range of salinities, temperatures, and pH values (Wittgens and Rosenau 2018). Most of these findings have been obtained using RLs extracted from *Pseudomonas aeruginosa* (i.e., predominantly Rha-Rha-C10-C10), an opportunistic pathogen in humans, animals, and plants. Some large-scale broths applications, containing RL producers, are generally weakened for safety-related concerns (Victor et al. 2019; Gutiérrez-Gómez et al. 2019; Chebbi et al. 2021). Accordingly, recent methodologies are starting to focus on nonpathogenic RLs’ producers, such as some *Burkholderia* spp. (Chong and Li 2017).

Given the CE’s fundamental principles, our study aims at producing RLs, using nonpathogenic producers towards the valorization of selected agricultural residues (i.e., nonfermented and fermented grape marcs, wine lees, and olive mill pomace). To the best of our knowledge, our study indicated for the first time that a nonpathogenic bacterium is able to producing long-chain rhamnolipids in MSM medium supplemented with winery residues, as sole carbon and energy source. The chemical structures of RLs congeners were also determined using UPLC-MS on those substrates for the first time in literature.

## Materials and methods

### Chemicals and agricultural residues

All the chemicals in our experiments, including glycerol (Glyc) (C_3_H_8_O_3_), ethyl acetate (C_4_H_8_O2), methanol (MeOH), chloroform (CH_3_Cl), sodium hydroxide (NaOH), hydrochloric acid (HCl), sulfuric acid (H_2_SO_4_), sodium chloride (NaCl), Tween 80, and Tween 20 were purchased from Sigma-Aldrich Company. For the emulsification index (E_24_), diesel oil was purchased from a nearby local fuel station in Milan. For agricultural wastes, nonfermented and fermented grape marcs (NF and F), wine lees (WL), and olive mill pomace (OMP) residues were obtained by local farmers in the Lombardy region during September 2019 within the CREIAMO project framework. All studied agricultural residues were stored at (−20 °C) for further conserving their physic-ochemical properties to chemical and biodiversity studies. Grapeseed oil, used as another model for winery wastes, was purchased from Aromatika.

### Physico-chemical characterization of agricultural residues

The physico-chemical characterizations of winery, i.e., nonfermented grape marcs, fermented grape marcs, wine lees, and olive oil residue, i.e., olive mill pomace were performed in collaboration with the Fondazione Edmund Mach, (https://www.fmach.it/). The measurements were determined based on the methods as follows: C:N (PDP 1042: 1997 Rev.0); protein (method for elementary analysis (nitrogen × 6.25)); pH (OIV-MA-AS313-15-R2011), ash (OIV-MA-AS2-04-R2009), alcoholic grade (DM12 March 1986 all. II-oenological by-products); total polyphenol content (TPC) (g/kg) (PDP 5001:1997 Rev. 0 (resasurin folin-Ciocalteu, spectrophotometric method)); malic acid (g/kg) (PDP 3086: 1997 Rev. 0 (ion chromatography)); lactic acid (g/kg) (OIV-MA-AS313-04 R2009 HPLC-UV method); tartaric acid(g/kg) (PDP 3086: 1997 Rev. 0 (ion chromatography)); lactic acid (g/kg); acetic acid (g/kg) (PDP 3061: 1997 Rev. 0 (HPLC-RI method); glycerin(g/kg) (PDP 3061: 1997 Rev. 0 (HPLC-RI method)); arabinose (g/kg), fructose (g/kg); galactose (g/kg), glucose (g/kg), mannose (g/kg), rhamnose (g/kg), ribose (g/kg), saccharose (g/kg), trehalose (g/kg), and xylose (g/kg) (PDP 3057: 1997 Rev. 0 (HPLC-PAD method)); total nitrogen (DM 13/09/1999 SO n° 185 GU n° 248 21/10/1999 Met XIV. 1 (method for elementary analysis)); total acidity (AOAC 942.15 (titration method)); calcium (mg/kg), phosphorus (mg/kg), magnesium (mg/kg), potassium (mg/kg) (PDP 3084: 2018 Rev. 10 (ICP-OES method)).

### Bacteria and growth monitoring

*Burkholderia thailandensis* E264 (DSM 13276), *Pseudomonas chlororaphis* (DSM 50083), and *Paraburkholderia kururiensis* KP23 (DSM 13646) were purchased from by Leibniz Institute DSMZ (German Collection of Microorganisms and Cell Cultures). All strains were routinely grown in Nutrient Broth (NB) agar plates and incubated at 30 °C and stored at 4 °C or cryopreserved at −20 °C and −80 °C supplemented with glycerol (Glyc) at (20%, v/v). For the biosurfactant production, an inoculum size of 3% (v/v) of preculture of the obtained cell suspension was inoculated in 250-ml Erlenmeyer flasks, containing the pre-sterilized agricultural broth. All the bacterial cultures were monitored for (8–10) days of incubation at 30 °C and 150 rpm, by determining the optical density at 600 nm, surface tension reduction (mN/m), E_24_, and dry cell biomass (g/L). Bacterial growth was assessed by measuring the optical density at 600 nm with Ultrospec 3000 UV/visible spectrophotometer (Pharmacia Biotech). A standard curve was also used to readily convert optical density values (OD_600 nm_) to the corresponding dry cell biomass concentration (g/L). A 10% series dilution was prepared using a *B. thailandensis* E264 cell culture grown up in 100 ml of NB + glycerol (Glyc) (4%,v/v) for 96 h; the cell culture was divided into two 50-ml aliquots, and OD_600 nm_ values were recorded for each 50-ml of solution before being centrifuged at 8000 *g* for 15 min (Funston et al. 2016). The resulting supernatants were discarded. The pellet formed after centrifugation was used to determine the dry cell weight (DCW) by weighing the pellet before and after drying at 80 °C until a constant weight was recorded. Dry cell weights were used along with their corresponding OD_600 nm_ values to generate a standard curve (*R*^2^ = 0.98; *Y* = 0.2528 × *X* + 0.2676).

### Media

The composition of the mineral salt medium (MSM) used in this study prepared in distilled water contained (in g/L): 2 NaNO_3_, 0.9 Na_2_HPO_4_, 0.7 KH_2_PO_4_, 0.4 MgSO_4_·7H_2_O, 0.1 CaCl_2_·2H_2_O, 0.001 FeSO_4_·7H_2_O; 1 ml of trace element solution (g/L): 0.7 ZnSO_4_·7H_2_O, 0.5 CuSO_4_·5H_2_O, 0.5 MnSO_4_H_2_O, 0.26 H_3_BO_4_, 0.06 Na_2_MoO_4_·2H_2_O (Chebbi et al. 2017a). The pH was adjusted to 7.4–7.6 with NaOH (5 M) and HCl (6 N) solutions before autoclaving for 30 min at 121 °C. Nutrient broth (NB), purchased from Sigma, was used for bacterial growth, consisting of peptone (15 g/L), yeast extract (3 g/L), sodium chloride (6 g/L), and glucose (1 g/L). The solid medium was prepared by adding agar at 15 g/L before autoclaving. Stock solutions of glucose, fructose, arabinose, galactose, lactose, xylose, and sucrose (20%, w/v) were sterilized through 0.45-μm sterile filters and kept away from light at +4 °C. To obtain a sugar concentration of 2% (w/v) in each bacterial culture, a volume of 10 ml of these sterile sugars solution was added in different 250-ml Erlenmeyer flasks containing 90 ml of sterile MSM medium. An inoculum proportion of 3% (v/v) of the seed culture was then added to the fresh media and incubated at 30 °C and 150 rpm for (8–10) days. For validating the biosurfactant activity, biotic and abiotic controls were prepared, incubated at the same conditions, and monitored over time. An inoculum size of 3% (v/v) was added to 100 ml of sterile MSM (biotic control with cells) and 100 ml of sterile NB + 4% v/v of Glyc (positive control), respectively.

### Winery residues: nonfermented and fermented grape marcs, wine lees

Winery residues, including nonfermented and fermented grape marcs (NF and F), wine lees (FC), were subjected to a pretreatment according to the methods described elsewhere, with slight modifications (Rivera et al. 2007). NF, F, and FC were agitated for 30 min in distilled water using a liquid/solid ratio of 10 g/g. The obtained broth was then filtered using Whatman paper. Various ratios of the liquors (v/v) were used as fermentation media. For comparing the results, the filtrated liquors from each sample were collected and added at different proportions (v/v) directly to 250-ml Erlenmeyer flasks containing MSM media and, therefore, sterilized at 121 °C for 30 min. Acid hydrolysis of NF, F, and FC was carried out by autoclaving at 121 °C in the presence of 3% H_2_SO_4_ (v/v) acid for 30 min using a liquid/solid ratio of 10 g/g. After the treatment, the hydrolysates were detoxified by adsorption with activated charcoal pellets (ultra carbon bios) using a liquid/solid ratio of 20 g/g and stirred at room temperature (25 °C) for 1 h. The activated charcoal was previously activated with hot water and dried at room temperature (25 °C) overnight. The detoxified hydrolysates were then filtered with Whatman paper, and the liquors neutralized with powder CaCO_3_ to a pH of 6.0 and finally adjusted to a final pH of 7.4–7.6 with 5 M NaOH. The CaSO_4_ precipitate was separated from the supernatant by filtration, and the clarified liquors were added directly to 250-ml Erlenmeyer flasks containing MSM medium. Then, the flasks were sterilized at 121 °C for 30 min. For both evaluating the biosurfactant production and the release of toxic components after acid pretreatment, yeast extract (3 g/L) (YE) and peptone (15 g/L) (PT) were added to the media NF, F, and FC.

### Olive oil residues: olive pomace

Three procedures were tested for olive mill pomace (OMP) residues to obtain increased cell growth and surface tension reduction. OMP was vigorously shacked for 30 min using a liquid/solid ratio of 10 g/g, left to decanting for 20–30 min, and the supernatant was collected and filtrate with 0.45 μm. An inoculum proportion of (2–5%) (v/v) of the filtrated OMP was added directly to sterilized MSM media and used for fermentation. For both evaluating the biosurfactant production and waste toxicity, yeast extract (3 g/L) and peptone (15 g/L) were added to the media in the presence of the OMP. An inoculum size of 2% and 5% (w/v) of OMP was added directly in MSM media and autoclaved, therefore, inoculated with the bacterial inoculum size of 3% (v/v). Due to the specific properties of oily agricultural residues, the three studied strains were re-screened on OMP as substrates for bacterial growth. For olive pomace: thermal and acid treatment, acid hydrolysis of OMP was carried out by autoclaving at 121 °C, in the presence and absence, of 3% H_2_SO_4_ (v/v) acid for 30 min using a liquid/solid ratio of 10 g/g. The hydrolysate was then filtered with Whatman paper, and the liquors were neutralized with NaOH (5 M) to a final pH of 7.3–7.4. Then, 3% (v/v) from thermal and acid-treated OMP were added to MSM medium and sterilized in an autoclave at 121 °C for 30 min.

### Surface tension determination and emulsification index

For evaluating the biosurfactant activity, the measurements of surface tension reduction were performed by a KRUSS K8 GmBH (Germany) tensiometer using the ring method. A volume of 10 ml from cell culture samples was taken and centrifuged at 8000 rpm for 15 min to remove cell biomass, and then the surface tension was measured. RLs’ ability to form emulsions was determined using the cell-free supernatants of the samples obtained during the fermentation. A volume of 3 ml of cell-free supernatant was added into a glass tube containing an equal volume (3.0 ml) of diesel oil. The tube was vortexed at high speed for 1 min and left to stand for 24 h at room temperature. The E_24_ was calculated by dividing the height of the emulsion layer (*he*) by the total height of the mixture (*hT*) and multiplying by 100. The estimation was carried out in triplicates. The emulsification activity was compared with that of chemical surfactants (i.e., Tween 20 and Tween 80) at 1 CMC and 2 CMC concentrations.

### Total sugars estimations by orcinol test

The orcinol test was performed for assessing the total sugars contents during the bacterial fermentation on agricultural residues. To each 100 μL sample, 900 μL of a solution containing 0.19% (w/v) orcinol (Sigma-Aldrich) in 53% H_2_SO4 (Sigma-Aldrich) was added. After, the samples were cooled at room temperature and the absorbances were measured at 421 nm. The concentrations of glycolipids (or sugars) present in the samples were calculated to those generated using a standard of rhamnose at concentrations of 0–2 g/L (*R*^2^ = 0.90). The total sugar degradation was expressed in percentage compared to abiotic controls (nonfermented winery residues), which are incubated at the same conditions.

### Rhamnolipid extraction, purification, and characterization: solid-phase extraction, CMC determination, study of the effect of pH, temperature, and salinity on biosurfactant stability

The biosurfactant extraction was carried out according to the method described by Smyth and Perfumo (Smyth and Perfumo 2010) with slight modifications. The cell-free supernatant was collected, centrifuged at 8000 rpm for 15 min for removing biomass, acidified to pH 2.5 with diluted HCl 40% (v/v), and then extracted three times with an equal volume of ethyl acetate. The organic phase was collected and dried by adding 0.1 g MgSO_4_ per 100 ml ethyl acetate (Perfumo et al. 2010; Chebbi et al. 2017a). A rotavapor (BṺCHI Rotavapor R-114) at 45 °C was used to evaporate the organic part and obtain a crude RLs extract. All crude extracts were measured gravimetrically before and after purification and expressed as mg/L. Solid-phase extraction (SPE) was used by StrataSI-1 Silica 55 μm, 70A column (Phenomenex), allowing the removal of any impurities from the crude RL extract. After washing the sample with chloroform, a solvent mixture of chloroform and methanol at a ratio of (1:1, v/v) (CHCl_3_:CH_4_O) has been used to elute the RLs. All eluted fractions were collected, dried, and then weighed. The CMC was estimated by measuring the surface tension of a series of concentrations of the RLs dissolved in distilled water with the pH adjusted to 7.1. The critical micelle concentration (CMC) of the crude biosurfactant was determined by measuring the surface tension at different concentrations. Crude biosurfactant has been diluted in milli-Q water at concentrations ranging from (0–2000 mg/L), and the CMC was determined by measuring the surface tension until a constant value was obtained. The cell-free supernatant was obtained by centrifugation at 8000 rpm after 120 h of incubation of E264 strain in NB + 4% (v/v) Glyc and was exposed to different pH (pH 2 to pH 13), at various concentrations of NaCl (from 6 to 100 g/L) and different temperatures (from −20 °C to 100 °C). Then, the surface tension was measured immediately for pH and salinity tests. After overnight incubation for temperatures ranging from −20 to 40 °C, the surface tension stability was determined (after 1 h for the measurements at 70 °C and 100 °C). The E_24_ was also determined for selected conditions.

### Characterization of rhamnolipids congeners using ultra performance liquid chromatography–tandem mass spectrometer on winery and olive oil residues

For UPLC separation (HClass, Waters), the following parameters were used: static phase, HSS T3 UPLC column (1.8 μm, 2.1 × 100 mm column (Waters, USA). Mobile phase 1: H_2_O (4 mM ammonium acetate), and mobile phase 2: MeCN, were used for chromatographic separation as follows: 0–12.2 min, 50–10% mobile phase 2; 12.24–12.6 min, 30–100% mobile phase 2; 12.60–12.9 min, 30–100% mobile phase 2; 12.96–20 min, 50–10-min mobile phase 2. The main conditions were as follows: flow rate 0.5 mL/min; volume injected, 10 μL; column temperature, 40 °C; sample temperature, 15 °C; run time, 20 min. The MS instrument (Acquity QDa, Waters) set up at 15 cone voltage for detecting in SIR (selected ion recording) the main predominant RLs according to the literature (Rha, rhamnose; Rha-Rha-C_14_C_14_, 761 m/z; Rha-Rha-C_12_-C_14_, 733 m/z; Rha-Rha-C_14_-C_16_, 789 m/z; Rha-C_14_-C_16_, 587 m/z; Rha-C_14_-C_14_, 615 m/z) (Funston et al. 2016). SPE purification was carried out using Starta SI-1 (55 μm, 70 A) 2 g/12 ml giga tubes. The samples were dissolved in ethyl acetate and added to the column, and pure CHCl_3_ was run thoroughly to clean unwanted products from the samples. Finally, the purified RL was eluted using a 1:1 v/v solution (CHCl_3_:CH_4_O).

## Results

### Physico-chemical characterization of winery and olive oil wastes

The physico-chemical characterizations of winery wastes have been carried out for the residues including, nonfermented grape marc (NF), fermented grape marc (F), and wine lees (WL) and olive mill pomace (OMP) (Tables 1, 2). The NF and F are characterized by low pH values of 4.0 and 3.6, respectively. A higher protein content was noted for NF at 47% in comparison to other winery wastes. NF is mainly characterized by high fructose and glucose contents, reaching more than 60 g/kg (Table 1). This finding is in agreement with prior studies showing that NF (white) marcs mainly consisted of soluble carbohydrates (i.e., fructose and glucose) for approximately 37.6% (w/w). In comparison, the fermented grape marcs (red) have a much lower percentage of soluble carbohydrates (4.6%, w/w) and a higher proportion of insoluble cell wall polysaccharides (53% of total carbohydrates measured) compared to white marcs (Corbin et al. 2015). At the same time, an important alcoholic grade of 7.3 ml/100 g was noted for NF residues. NF and F are also well described by the tartaric content, which is another valuable natural product for other valorizing strategies of these potential agricultural wastes (Rodríguez et al. 2010) (Table 1). As a result of agro-industrial activities in Europe, a great number of residues are generated; among them, olive pomace stands out as one of the most abundant. First, it was characterized by a pH of 4.95 (Table 2). A relatively higher total acidity was also noted at around 5.0 g/kg, which is in agreement with prior studies (Miranda et al. 2012). The calcium, phosphorus, and potassium contents are also highly abundant in this agricultural residue. Those oligo-elements could be very beneficial for increasing the biosurfactant production by some microorganisms (Portilla et al. 2008). Whereas, in comparison to the winery residues, olive mill pomace contains lower sugar contents (Table 2).

**Table 1:** Physico-chemical characterization of the studied agricultural residues

	Nonfermented grape marc (NF)	Fermented grape marc (F)	Wine lees (WL)
pH	4.01	3.6	5.9
Protein (g/kg)	39	34	47
Loss on drying at 100–105 °C (%)	62.53	74.52	66.54
C/N ratio	30.6	25.1	24.5
Ash (%)	4.94	8.18	5.31
Alcoholic grade (ml/100 g)	0.7	7.3	0.2
Total polyphenol content (TPC) (g/kg)	6.7	6.8	6.3
Malic acid (g/kg)	4.4	1.9	n.q (<0.1 g/kg)
Lactic acid (g/kg)	n.q (<0.1)	n.q (<0.1 g/kg)	n.q (<0.1 g/kg)
Tartaric acid (g/kg)	14.0	22.3	0.8
Acetic acid (g/kg)	1.23	0.71	0.79
Glycerin (g/kg)	4.39	5.35	0.35
Arabinose (g/kg)	n.q (<0.1)	0.3	n.q (<0.1 g/kg)
Fructose (g/kg)	61.3	0.2	0.3
Galactose (g/kg)	0.1	n.q (<0.1 g/kg)	n.q (<0.1 g/kg)
Glucose (g/kg)	65.4	0.4 g/kg	0.3
Mannose (g/kg)	n.q (<0.1 g/kg)	n.q (<0.1 g/kg)	n.q (<0.1 g/kg)
Rhamnose (g/kg)	n.q (<0.1 g/kg)	n.q (<0.1 g/kg)	n.q (<0.1 g/kg)
Ribose (g/kg)	n.q (<0.1 g/kg)	n.q (<0.1 g/kg)	n.q (<0.1 g/kg)
Saccharose (g/kg)	n.q (<0.1 g/kg)	n.q (<0.1 g/kg)	n.q (<0.1 g/kg)
Trehalose (g/kg)	n.q (<0.1 g/kg)	n.q (<0.1 g/kg)	n.q (<0.1 g/kg)
Xylose (g/kg)	n.q (<0.1 g/kg)	n.q (<0.1 g/kg)	n.q (<0.1 g/kg)

*n.q*, not quantifiable

**Table 2:** Physico-chemical characterization of olive mill pomace

	Olive oil pomace (OMP)
pH	4.95
Total nitrogen (%)	0.93
Loss on drying ( 100-105 °C) (%)	56.94
Total acidity (g/kg)	5.0
Ash (%)	2.79
Calcium (mg/kg)	1025
Phosphorous (mg/kg)	340
Magnesium (mg/kg)	158
Potassium (mg/kg)	4615
Total polyphenolic (g/kg)	1.1
Glycerin (g/kg)	0.82
Fructose (g/kg)	n.q (<0.1 g/kg)
Glucose (g/kg)	0.2
Arabinose (g/kg)	n.q (<0.1 g/kg)
Galactose (g/kg)	0.2
Mannose (g/kg)	n.q (<0.1 g/kg)
Ramnose (g/kg)	n.q (<0.1 g/kg)
Ribose (g/kg)	n.q (<0.1 g/kg)
Saccharose (g/kg)	n.q (<0.1 g/kg)
Trealose (g/kg)	n.q (<0.1 g/kg)
Xylose (g/kg)	n.q (<0.1 g/kg)

*n.q*, not quantifiable

### Screening of a nonpathogenic rhamnolipid producer

**Fig. 1:**
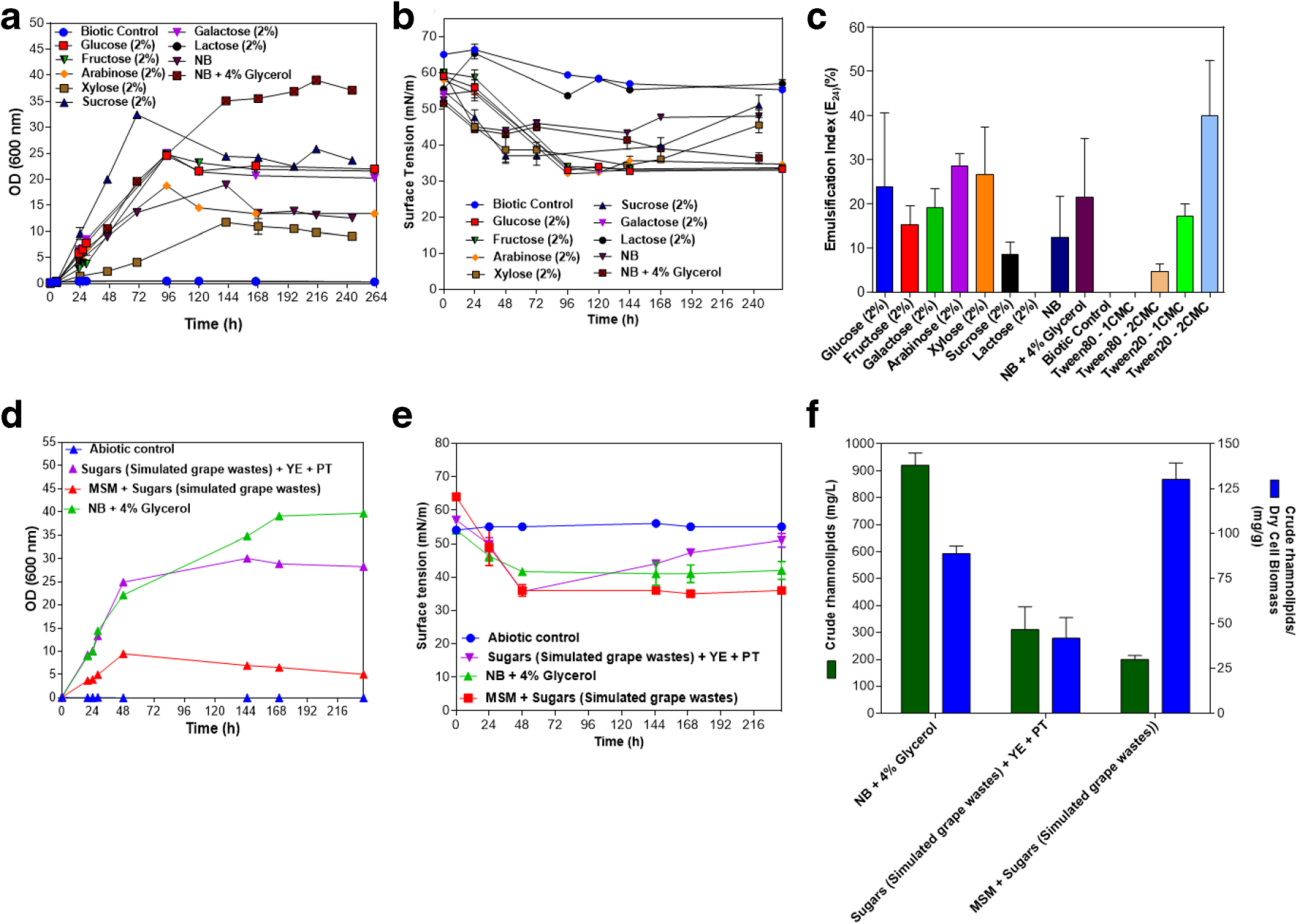
**A** Monitoring the cell growth (OD 600 nm), **B** surface tension reduction (mN/m), and **C** the emulsifying capacity (at 144 h of incubation) (%) of the strain *B. thailandensis* E264 after incubation on various carbon sources, as sole carbon and energy sources in MSM medium, during 263 h at 30 °C and 150 rpm. **C** The emulsifying potential of the cell-free supernatant using sugars as carbon sources in MSM medium at 144 h of incubation. **D** Growth monitoring of the strain E264 and **E** surface tension reduction on sugars (simulated grapes wastes medium), in the presence and absence yeast extract and peptone and the **F** crude rhamnolipid yields (mg/L), after 236 h of incubation at 30 °C and 150 rpm. NB, nutrient broth; MSM, mineral salt medium; YE, yeast extract (0.3%, w/v); PE, peptone (1.5%, w/v), sugars (simulated grapes wastes medium): 2.3 g/L glucose, 4.7 g/L xylose, 1.8 g/L galactose 0.19 g/L fructose

The pathogenic character of RLs producers, including the most studied strain *Pseudomonas aeruginosa*, is considered one of the major reasons for considering other safe RL producers (Victor et al. 2019). In our study, *B.thailandensis* E264 (Elshikh et al. 2017), *P. chlororaphis* DSM 50083 (Gunther IV et al. 2005), *P. kururiensis* DSM 13646 (Tavares et al. 2013) were selected for conducting the present study, due to their promising properties and abilities to produce RLs with a nonpathogenic character. Based on grape marcs’ main components, a screening step on several model sugars (i.e., fructose, galactose, arabinose, xylose, sucrose, galactose, lactose) at 2% (w/v) was performed for the three strains at 30 °C and 150 rpm. For that, over the incubation time, we evaluated the cell growth (OD 600 nm), the reduction of surface tension, and the E_24_ at 30 °C and 150 rpm (Fig. 1, S1). *P. chlororaphis* DSM 500083 and *P. kururiensis* DSM 13646 showed the ability to metabolize different sugars (e.g., glucose, fructose, galactose) as sole carbon and energy source at 30 °C and 180 rpm, but no reductions of surface tension were detected in cell-free supernatants during 192 h of incubation (Fig. S1). The strain *P. kururiensis* DSM 13646 was found not able to significantly reduce the surface tension on all of the sugars tested, despite the growth on several sugars, as sole carbon and energy sources (i.e., glucose, fructose, arabinose, xylose, galactose), in MSM medium at 30 °C and 150 rpm (Fig. S1). No growth was also noted for sucrose and lactose incubated at the same conditions.

The cell growth (OD 600 nm), surface tension reduction (mN/m), and E_24_ (%) were monitored for the strain *B. thailandensis* E264 on MSM medium supplemented with sugars at (2%,w/v), as sole carbon and energy sources, during 236 h of incubation at 30 °C and 150 rpm (Fig. 1A, B, C, D). The strain E264 grew on numerous sugars, including glucose, fructose, galactose, arabinose, xylose, sucrose, as sole carbon and energy sources (Fig. 1A), while it was not able to grow on lactose. Interestingly, in comparison to abiotic and biotic controls, strain E264 displayed a high capacity to reduce the surface tension of cell-free supernatants to an average of 33–36 mN/m when growing on glucose, fructose, galactose, arabinose, xylose, and sucrose (Fig. 1B). The E_24_ was quite similar to that of chemical surfactants, such as Tween 20 and Tween 80 (Fig. 1C). After that, the yields of RLs were also evaluated on three controls; (1) NB + 4% Glyc, (2) sugars (simulated grape wastes) + YE 0.3% (w/v), +PT1.5% (w/v), (3) MSM+ sugars (simulated grape marc), after incubation during 263 h at 30 °C and 150 rpm (Fig. 1F), after 236 h of incubation at 30 °C and 150 rpm. The production of crude RLs of the strain E264 was as follows: 920 mg/L (±46) (89 mg/g biomass ±4), 310 mg/L (±60) (42 mg/g biomass ±8.1), and 200 mg/L (±10) (130 mg/g biomass ±6.5), when growing on (1), (2), and (3), respectively (Fig. 1D, E, F). On NB + 4% Glyc, this bacterium exhibited the highest yield of crude RLs compared to those obtained on two other media compositions. However, the growth was not correlated with the RL yield of production.

### Production of rhamnolipid on winery residues: raw nonfermented and fermented grape marcs

**Fig. 2:**
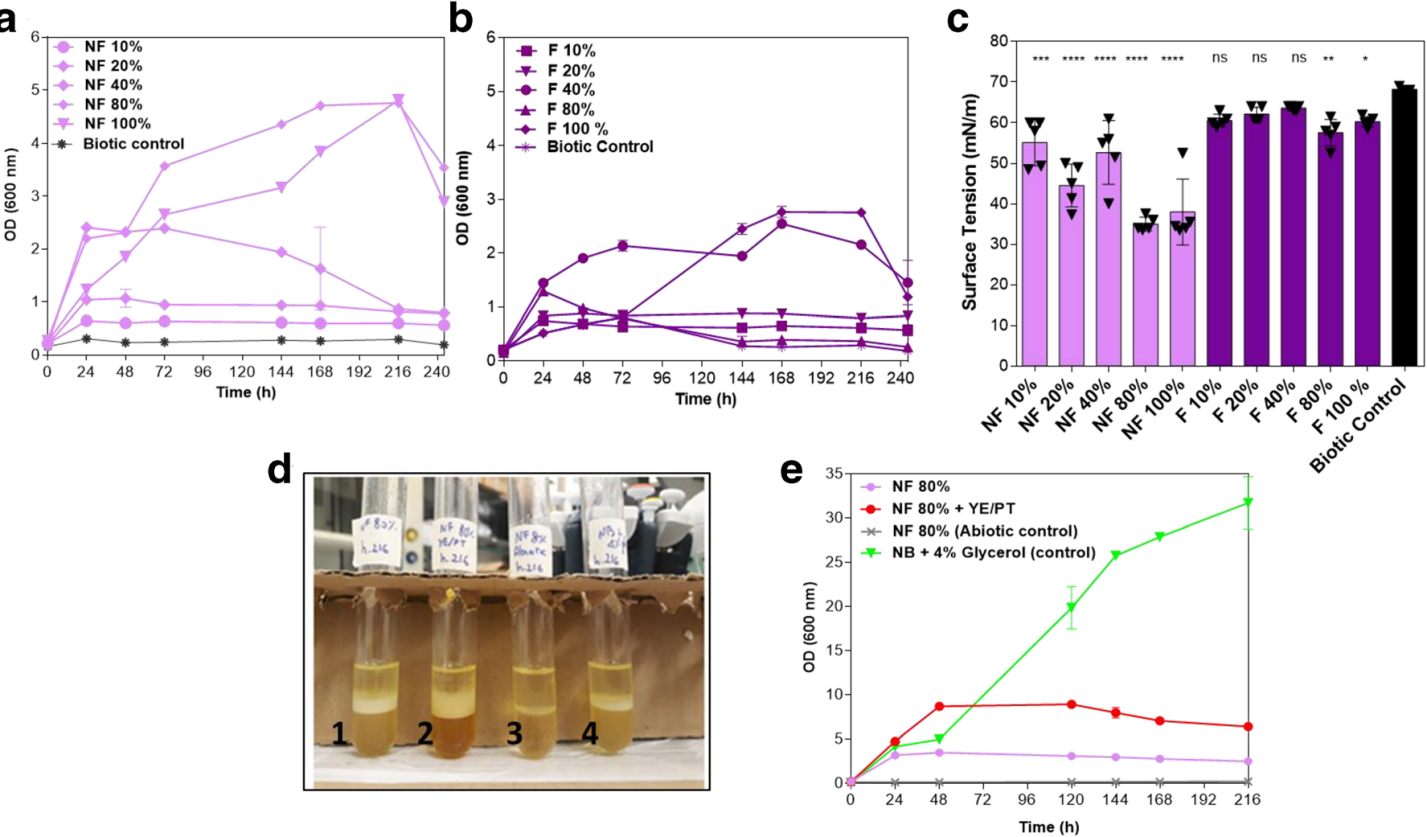
(**A**) Monitoring the cell growth (OD 600 nm) and (**B**) the surface tension reduction (mN/m) of *B. thailandensis* E264, (**C**) in the presence of nonfermented grape marcs (NF) and fermented grape marcs (F) fractions, in MSM medium during 240 h at 30 °C and 150 rpm. (**p < 0.05), ****(*p* < 0.01) (compared to biotic controls). **E**, **D** Emulsification potential of cell-free supernatant on NF-fermented grape marcs 80% (v/v), 80% (v/v) + YE/PT, and NB +4% glycerol (Glyc) (v/v) and chemical control (NB 80%). YE, yeast extract (0.3%, w/v); PE, peptone (1.5%, w/v)

In this study, we assessed numerous chemical and physical treatments of various conditions of winery and olive oil residues, including raw, thermal, thermal + detoxification using coal with taking into account the cost of each step. We observed that the combined acidic thermal of grape marcs and lees treatments generated highly colored liquors, which are very challenging for monitoring bacterial growth during the time without any further chemical (or physical) purification steps due to their possible contents of other toxic metabolites after the hydrolysis. In the raw condition, the cell growth of strain E264 and surface tension reductions were surveyed in MSM medium supplemented with increasing fractions of raw purified liquors of nonfermented grape marcs (NF) and fermented grape marcs (F) (Fig. 2A, B). As shown in Fig. 2, the strain E264 was found to grow well at higher fractions of NF grape marcs liquor up to 80% and 100% (v/v) and reducing the surface tension to around 35 mN/m of the cell-free supernatants, at 30 and 150 rpm. Conversely, for fermented grape marcs (F), no significant difference was recorded in the reduction of surface tension compared to that of biotic and abiotic controls, incubated at the same conditions (*p* < 0.05) (Fig. 1C). Given those preliminary results, the percentage of NF grape marcs liquor was fixed at 80% (v/v) for the RLs’ production, as sole carbon and energy source, in MSM at 30 °C and 150 rpm. Besides, the fermentation of strain E264 on winery residues showed a substantial foaming and emulsification capacity, commonly associated with RLs production (Fig. S2). In the presence of NB + 4% Gly, a substantial biomass growth was recorded but a less reduction of surface tension (Fig. S2). It was also observed that the addition of yeast extract and peptone increased the emulsifying potential (on diesel as a substrate) but was not correlated with the reduction of surface tension during the incubation time (Fig. S2). A brownish color, generally characterizing RLs, was noted to be clearer for the biosurfactant extracted on the condition of NF 80% (v/v), indicating a higher purity of those produced glycolipids (Fig. 2H). The consumption of sugars was also monitored using the orcinol method (Fig. S3). It was observed that the sugars’ degradation significantly decreased after 48 h in all of the samples tested (Fig. S3). The orcinol confirmed the degradation of carbohydrates in those agricultural residues compared to abiotic controls (Fig. S3). In our results, the rapid surface tension reduction on NF 80% (v/v), as sole carbon and energy source, can be explained by a rapid metabolism of the sugars, and then the bacterium immediately entered into the stationary phase, which is associated with RL production (Fig. 2C and S3). For NF 80% (v/v) and NB + 4% Glyc, the surface tension remained stable during the incubation time, whereas the NF 80% (v/v) + YE/PT increased to 57 mN/m at the end of fermentation (Fig. S2).

### Production of rhamnolipid using olive oil residues as substrates: olive mill pomace

**Fig. 3:**
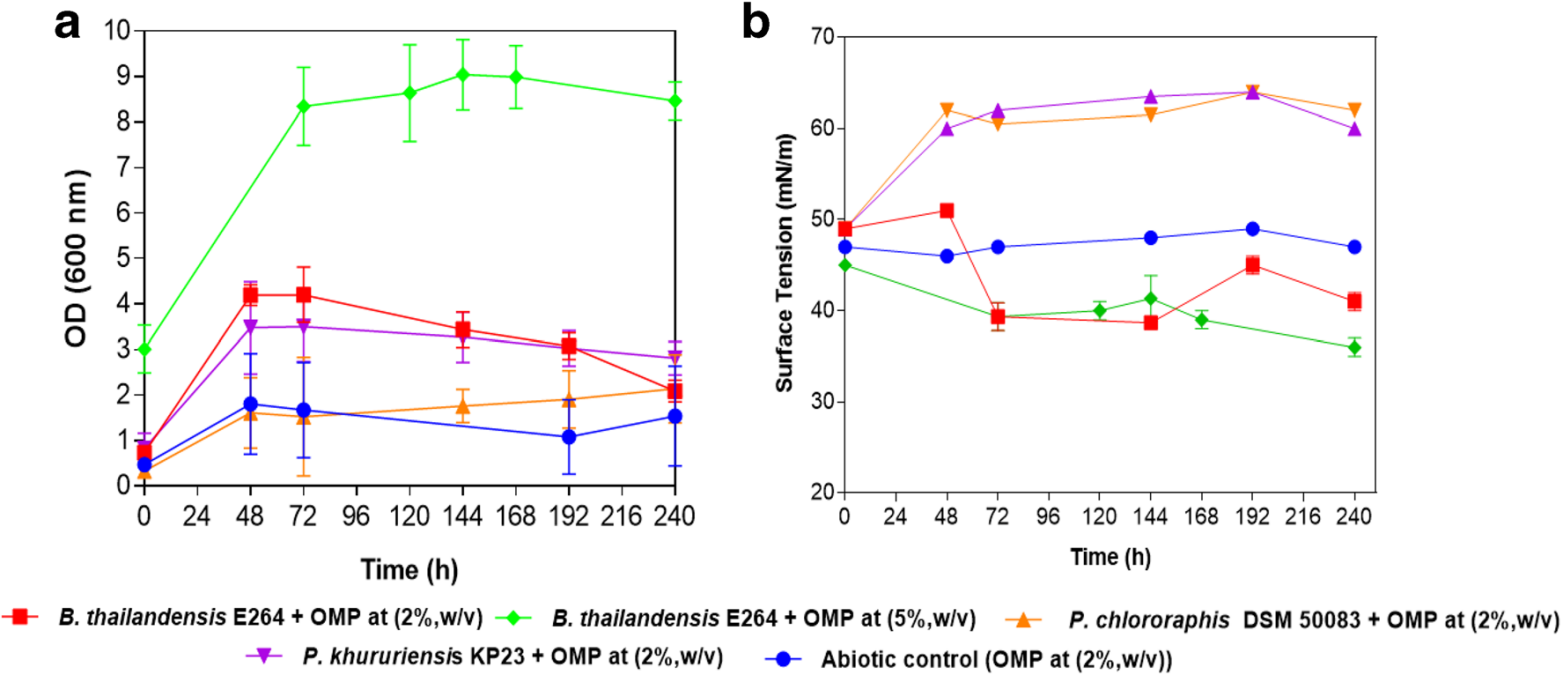
**A** Monitoring the growth of *B. thailandensis* E264, *P. chlororaphis*, and *P. kururiensis* (OD 600 nm) and surface tension reduction (mN/m) on olive mill pomace (OMP) at 30 °C and 150 rpm during 240 h (**A** and **B**)

Based on the different proprieties between the winery and olive oil residues, *B. thailandensis* E264, *P. chlororaphis* DSM 50083, and *P. kururiensis* DSM 13646 were reevaluated for their capacities to grow on olive mill pomace and reducing the surface tension on MSM medium, at 30 °C and 150 rpm, at 2% (w/v) and 5% (w/v) (Fig. 3AB). A significant reduction of surface tension was recorded to around 38 mN/m after 72 h for the E264 strain at 2% and 5% of olive OMP (w/v) (Fig. 3B). While for *P.chlororaphis* DSM 50083, and *P. kururiensis* DSM 13646 were not able to reduce surface tension, despite the growth recorded (Fig. 3). The strain E264 was also able to use olive pomace as the sole carbon and energy source and capable of reducing the surface tension during the incubation at 30 °C and 150 rpm (Fig. 3 AB, S4).

### Characterizing rhamnolipid produced by *B. thailandensis* E264: the critical micelle concentration and stability studies (pH, temperature, and salinity)

**Fig. 4:**
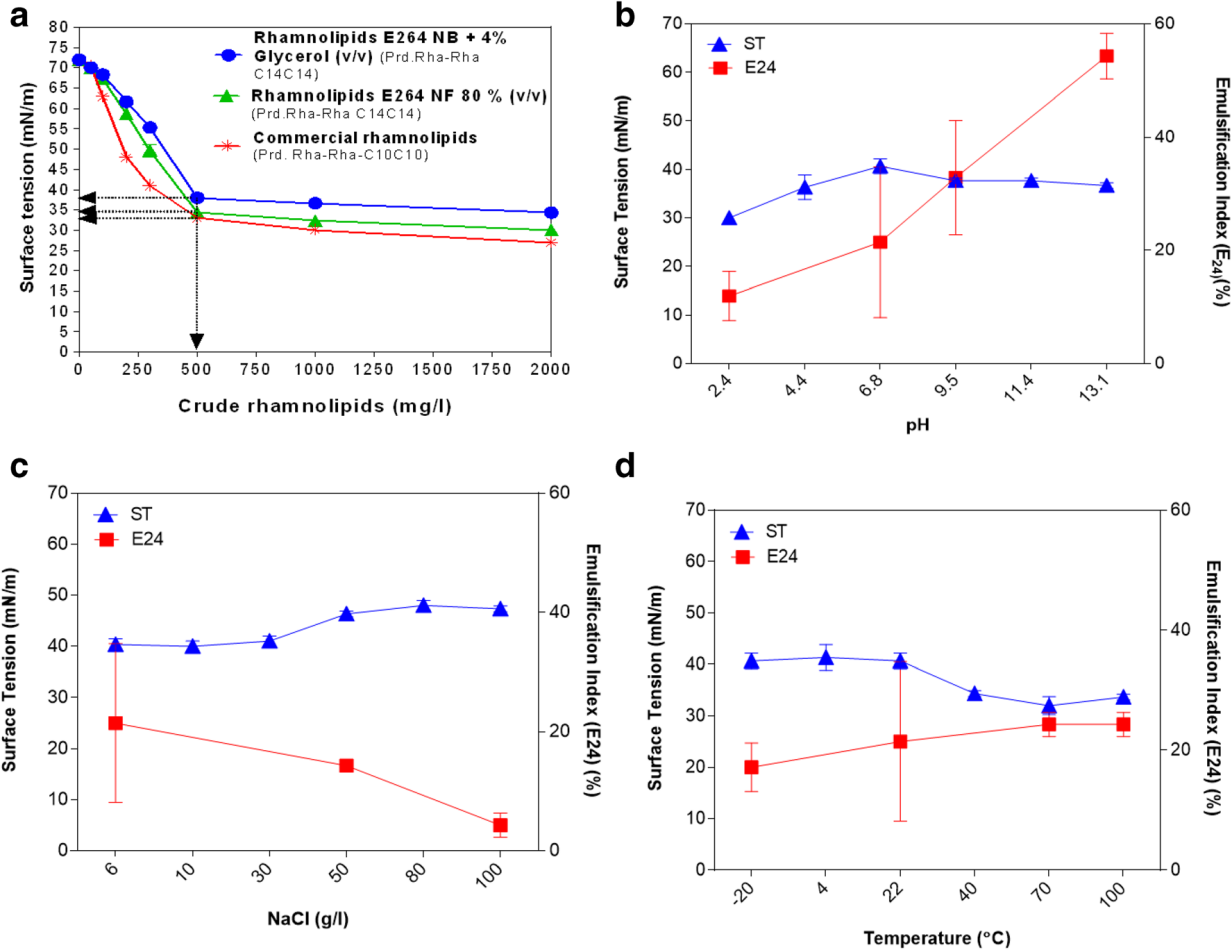
Determination of critical micelle concentration (CMC) (**A**); effect of emulsifying potential and surface tension reduction on pH (**B**), sodium chloride concentration (**C**), and temperature (**D**)

The critical micelle concentration (CMC) was determined for the long-chain RLs produced by the strain E264 (Fig. 4A). For the crude RLs extracted from NB + 4% Glyc, the surface tension decreased, reaching a minimum value of 38 mN/m, and was characterized by a CMC of 500 mg/L (Fig. 4). Conversely, on NF 80% extract, a similar CMC concentration and a higher surface tension decrease to around 34.6 mN/m (Fig. 4A) were observed. Interestingly, these results are comparable to those recorded on the commercial crude RLs (lower chain RL) (Prd. Rha-Rha-C_10_C_10_), showing a CMC of 500 mg/L and a surface tension value of 33 mN/m. To compare the purity of RL produced on NB + 4% an NB, we reevaluated the reduction of surface tension and the CMC values of the two extracted biosurfactants on agricultural residues (Fig. 4A). Both extracts have shown the same CMC around 500 mg/L, though the reduction of surface tension is slightly better, reaching around 34.5 mN/m (Fig. 4A). Furthermore, the effects of pH, salinity, and temperature on surface tension and emulsification activity of RLs produced biosurfactant produced by the strain E264 were also investigated (Fig. 4B, C, D). Its RLs displayed no significant variations on the surface tension of the cell-free supernatant between pH 6.8 (ST = 40.6 mN/m) and pH 13.1 (ST = 36.6 mN/m) (Fig. 4B), whereas the E_24_ gradually increased from 12% at pH 2.40, to 33% at pH 9.6, to 54% at pH 13.1 (Fig. 4B). For the salinity effects, the RLs from strain E264 were found to be affected above 50 g/L NaCl (ST = 46 mN/m, E_24_ = 14%) and significant changes were observed in E_24_ with addition of up to 100 g/L NaCl (ST = 49.5 mN/m, E_24_ = 4%) (Fig. 4C). Moreover, the produced RLs were also found to be thermostable over a wide range of temperatures (−20 to 100 °C) (Fig. 4D). The cell-free supernatant heating at 100 °C was found to slightly affect the emulsifying potential (ST = 33.6 mN/m, E_24_ = 24%) (Fig. 4D). The RLs produced by strain E264 showed a CMC of 500 mg/L and good stability capacity versus pH, temperature, and salinity (Fig. 4).

### Rhamnolipid yields on winery and olive oil residues and purification: purification with SPE

**Fig. 5:**
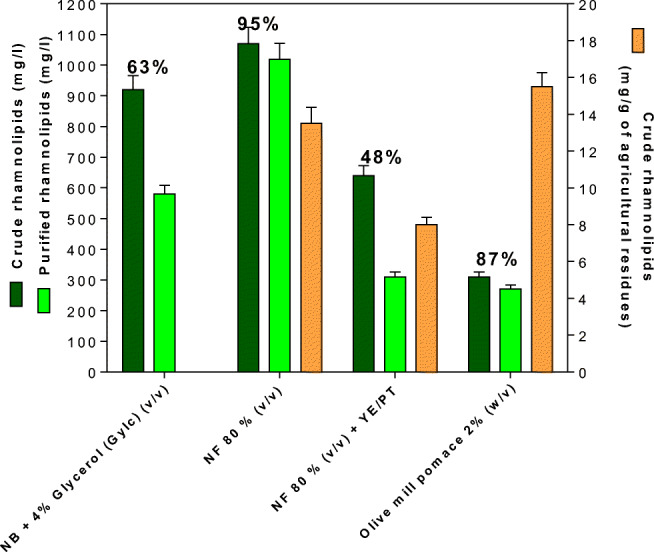
Crude and purified rhamnolipid yields (mg/) and percentage of purity of the crude extracts from *B. thailandensis* E264 fermentation on NF at 80% (v/v), NF 80 % (v/v) + YE/PT, olive mill pomace (OMP) at 2% (w/v), and NB + 4% glycerol (Glyc), after incubation during at 30 and 150 rpm

For RL purification, the crude biosurfactant produced on NB + 4% Glyc, NF 80%, NF 80% + yeast extract (YE) + peptone (PT), and olive mill pomace as a substrate were subjected to SPE chromatography (Fig. 5). SPE was also used to remove impurities from the sample before analysis (Fig. 5). After purification, NF 80% (v/v) condition showed a RL yield of 1020 mg/L, which represented up to 95% of purity, equivalent to 13.37 mg of crude RL per gram of NF grape marcs. Conversely, OMP at 2% (w/v) revealed a yield of 270 mg/L, which corresponded to 87% of the corresponding crude extract (15 mg/g agricultural residues). NB + 4 % Glyc showed a lower of around 63. Taking together, the RL samples, purified from NF 80% (v/v) and OMP 2% (w/v), were found to be the purest, showing a yield up to 95% and 87%, respectively (Fig. 5). The addition of yeast extract and peptone resulted in neither an increase in RL yields nor purity (Fig. 5). Raw nonfermented grape marcs and raw olive pomace could represent suitable agricultural substrates for biosurfactant production and by the direct use of these molecules as crude extracts (or even cell-free supernatants) for industrial applications. The strain E264 was found to produce around 310 mg/L of crude RLs after incubation for 144 h at 30 °C and 150 rpm at 2% (w/v) (Fig. 5). Another control was also used in this experiment, grape seed oil at 2% (v/v) in MSM medium during 216 h and 150 rpm at 30 °C, showing around 2750 mg/L of crude RLs with a purity of 68%.

### Separation of rhamnolipid congeners using UPLC-MS produced on nonfermented grape marcs and olive pomace

**Fig. 6:**
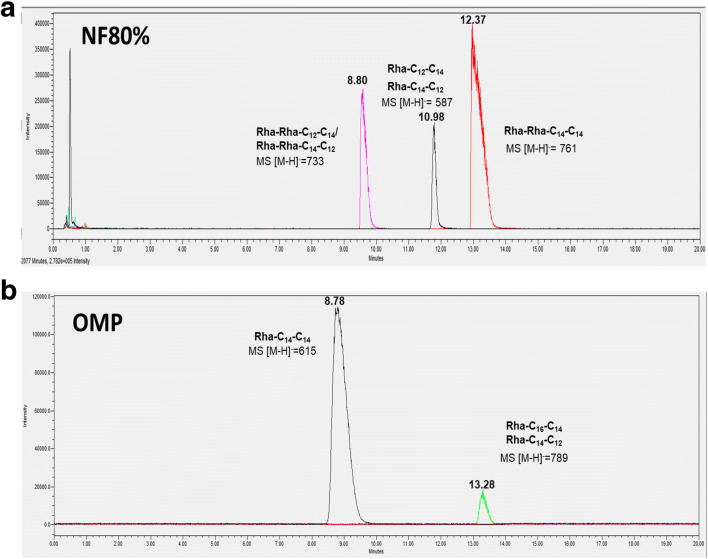
Chromatograms UPLC-MS in negative ion mode ionization of rhamnolipid after SPE purification produced on nonfermented grape marcs at 80% (v/v) in MSM medium (**A**) (after 216 h) and olive mil pomace (OMP) (**B**) (after 144 h), as sole carbon and energy sources, by *B. thailandensis* E264 in negative ion mode ionization at 30 °C and 150 rpm (The ions were selected by selected ion recording (SIR) based on predominant RLs produced by E264 (Funston et al. 2016))

To give a qualitative assessment of the RL congeners produced on NF grape marcs and olive pomace, UPLC-MS was applied to separate individual congeners present in the semi-purified extracts (Fig. 6, Table 3). For the NF grape marcs, the trace obtained from UPLC revealed a good separation of three congeners with the predominance of Rha-Rha-C_14_-C_14_ (MS[M-H = 761]) (Fig. 6A, Table 3). For the olive mill pomace, only two congeners were detected, i.e., Rha-C_14_-C_14_ and Rha-C_16-_C_14_/Rha-C_14_-C_16_ (Fig. 6B, Table 3). Accordingly, the variation of RL congeners produced by *B.thailendensis* E264 was found to be low; only six congeners produced on NB + 4% Glyc in significant quantities (Funston et al. 2016). The Di-RL C_14_C_14_ was the highest abundance (41.88%) followed by Di-RL C_12_-C_14_/C_14_-C_12_ (16.7%) and DI-RL C_14_-C_16_/C_16-_C_14_ (8.14%) (Funston et al. 2016).

**Table 3:** Relative abundance of specific rhamnolipid produced on nonfermented grape marcs (after 216 h) and olive mill pomace (after 144) in MSM medium, as sole carbon and energy sources, by strain E264 at 30 °C and 150 rpm

Agricultural wastes	Rhamnolipid congener	Pseudomolecular (m/z)	Retention time (min)	Relative abundance (%)
Nonfermented grape marcs (NF)	Rha-C_12_-C_14_/Rha-C_12_-C_14_	587	10.98	12.56
	ND	643	10.9	2.39
	Rha-Rha-C_12_-C_14_/Rha-Rha-C_12_	733	8.88	27.02
	Rha-Rha-C_14-_C_14_	761	12.37	53.16
	Total			100%
Olive mill pomace (OMP)	Rha-C_14-_C_14_	615	8.78	93.44
	Rha-C_16-_C_14_/Rha-C_14-_C_16_	789	13.28	6.55
	Total			100%

## Discussion

Our study aims to assess the rhamnolipid production from winery and olive oil residues as low-cost carbon sources by nonpathogenic strains. Our results indicated that the strain *B. thailandensis* E264 was a good candidate for further fermentation attempts on the winery wastes (Fig. 1). Previous works have reported biotechnological relevant traits of *Burkholderia* spp*.* Indeed, the type strain *P. kururiensis* KP23^T^ (JCM10599, formerly *B. kururiensis* KP23^T^) is a nonpathogenic environmental strain isolated from an aquifer in Japan, which possesses promising bioremediation capabilities (Zhang et al. 2000). *B. kururiensis* KP23^T^ has been described as a TCE-degrading bacterium and isolated from an aquifer at a TCE-polluted site in Japan (Zhang et al. 2000). The RL production by *B. kururiensis*, and its characterization by LTQ-Orbitrap hybrid mass spectrometry was shown, and the population of RLs produced by *B. kururiensis* KP23^T^ has been revealed molecular species commonly observed in *Pseudomonas* spp. and/or *Burkholderia* spp. (Tavares et al. 2013). Some studies have also noted that some strains belonging to the *Pseudomonas* sp*.* showed variabilities in the BS activity, and they suggested an advanced reclassification of all the RL producers (Victor et al. 2019). One possible explanation for the incapability to reduce the surface tension of these two previous strains could be the metabolic precursors, which could be shifted to other competing pathways or the activation of biosynthetic pathways under those conditions, leading to RL yields’ limitation (Wittgens and Rosenau 2018; Arnold et al. 2019). Besides, our results have also detected most of the predominant ions on those agricultural residues previously shown on *B. thailandensis* E264, where the NB medium has been used supplemented with 4% Glyc (Funston et al. 2016). Few *Burkholderia* genus members have been described in previous reports for RL production, though not using agricultural wastes as substrates. For instance, Hörmann et al. (2010) have reported the production of 0.045 g/L of Di-RLs (Rha-Rha-C_14_-C_14_) from *B. plantarii*, *B. kururiensis* KP23^T^ (Tavares et al. 2013), and *B. glumae* (Nitschke et al. 2011) have also been shown to produce 0.78, 1, and 1.7 g/L RLs respectively, with a major component of long-chain RLs C_14_-C_14_. Strain E264 has been described to have two identical genes containing copies of RL synthesis genes (*rhl*A, *rhl*B, *rhl*C) present at different sites in its genome (Dubeau et al. 2009). In contrast, *Pseudomonas aeruginosa* has only one copy of each gene with the *rhl*A and *rhl*B genes located in the same operon, while *rhl*C is located separately (Rahim et al. 2001). Another advantage of the present strain E264 is its classification as a biosafety level I or a putative non-pathogen, which makes it an attractive substitute for RL production, compared with *P. aeruginosa*, an opportunistic pathogen or biosafety level II organism (Victor et al. 2019).

To the best of our knowledge, our study indicated for the first time that a nonpathogenic bacterium and RL producer could reduce surface tension on winery residues, i.e., nonfermented grape marcs and, therefore, producing surface-active agents in MSM supplemented with those agricultural wastes (Fig. 2, S2). After the grapes are crushed and processed for winemaking, approximately 20% of the starting material remains as seed, skin, and pulp (Spanghero et al. 2009). The residual carbohydrates remaining in the marcs after the previous step are predominantly water-soluble monosaccharides, oligosaccharides, and polysaccharides, and water-insoluble structural polysaccharides from the cell wall (Corbin et al. 2015). The soluble carbohydrates can be extracted with minimal energetic input and may be directly used as a raw substrate for fermentation, whereas cell wall polysaccharides need to be released through pretreatment and saccharification (Corbin et al. 2015). Based on the physico-chemical characterization, for the nonfermented grape marc, the main predominant sugars are fructose (61.3 g/kg) and glucose (65.4 g/kg), which seem to be used as sole carbon and energy sources by the strain E264 in MSM medium (Table 1). It has been revealed that one-third of the dry weight (37.6%, w/w) and 70% of the total carbohydrate are water-soluble carbohydrates (WSC) in white marcs (Corbin et al. 2015). The grapes marcs’ compositions are directly linked to the characteristics of the original grapes, which depend both on extrinsic factors such as edaphoclimatic conditions and viticultural practices and intrinsic factors such as variety, maturity, and sanitary conditions (García-Lomillo and González-SanJosé 2017). There are differences between the unfermented and fermented grape marcs mainly in the residual sugar content: unfermented white marcs contain more residual sugars than the fermented one, while the fermented red marcs have low sugar content and some quantities of alcohol (ethanol) (Hixson et al. 2016). The soluble carbohydrates can be extracted with minimal energetic input and may be directly used as a raw substrate for fermentation, whereas cell wall polysaccharides need to be released through pretreatments (Corbin et al. 2015). Nonfermented (white) marcs mainly consisted of soluble carbohydrates (i.e., fructose and glucose) for approximately 37.6% (w/w), while the fermented grape marcs (red) has a much lower percentage of soluble carbohydrates (4.6%, w/w) and a higher proportion of insoluble cell wall polysaccharides (53% of total carbohydrates measured) compared to white marcs (Corbin et al. 2015). The comparatively low amount of soluble carbohydrates detected in the red fermented marcs may be due to the grapes’ processing during winemaking, as red marcs are left in contact with the must during the fermentation to enhance the color and sensory attributes of the wine (Baiano et al. 2016). The low quantity of residual sugars combined with the presence of by-products of alcoholic fermentation (reaching around 7.3 ml/100 ml) (e.g., organic acids, phenolic compounds) might justify the lower growth on fermented grape marcs compared to that obtained on nonfermented residues (Fig. 2 AB, Table 1). The ethanol in those winery residues also has a bactericidal activity by acting as a membrane disruptor, interfering with cell division, and affecting steady-state growth (Chatterjee et al. 2006). This could explain the absence of biosurfactant activity on that type of fermented grape marcs, which likely contain ethanol in addition to other metabolites. This could lead to further pretreatments’ steps to get rid these residues before biosurfactant production investigations. Based on the growth curve analysis, it is clear that E264 revealed a lower bacterial density using fractions of NF grape marcs, compared to that obtained on NB + 4% Glyc, and an extended stationary phase, which might be explained by the fact that most of the soil bacteria are adapted to survive throughout cell stress and nutrient starvation (Fig. 2, S2). This finding seems to be promising in terms of obtaining higher RL yields, resisting to the toxic chemicals contained in some agricultural residues after winemaking processing. This strain has been shown to coproduce the polyhydroxybutyrate (PHB) in the presence of specific substrates like the used corn oils, yielding larger size and number of bacterial cells due to its accumulation in the intracellular compartment (Kourmentza et al. 2018). It has been also demonstrated that the strain E264 showed higher yields of RLs at 25 °C compared to that obtained at 30 °C (Funston et al. 2017).

Concerning the vegetable oils’ related residues, the processing of the edible oil generates large amounts of wastes and by-products with a high content of fats, oils, and other compounds, including soap stocks, oilseed cakes, fatty acid residues, semisolid effluents, and water-soluble effluents (Makkar et al. 2011). These residues were widely considered as principal sources of both water and soil contamination due to their low degradability and even toxicity, e.g., used corn oil (Banat et al. 2014). The use of this type of wastes has been reported for biosurfactant production, mostly lipopeptides (Hentati et al. 2019). Like the winery agricultural residues, little knowledge is currently available concerning the production of microbial surface-active agents from olive mill pomace residues (Table 4). Mercadé et al. (1993) were the first group to show the RLs production of by a *Pseudomonas* sp., grown on olive oil mill effluent (OOME), as the sole carbon source, producing up to 0.058 g/g of the substrate with the use of 100 g/L of OOME and 2.5 g/L of NaNO_3_. While Ramirez et al. (Moya Ramírez et al. 2015) have used a solid waste called “Alperujo” resulting from the extraction of olive oil in Spain: this olive oil residue has been used to produce surfactin and RLs using *B. subtilis* N1 and *P. aeruginosa* PAO1, respectively*.* A maximum concentration of 3.12 mg/L with 2% w/v of the residue with *B. subtilis* and 8.78 mg/L of RL with 2% w/v increasing to 191.46 mg/L with 10% w/v using *P. aeruginosa* (Moya Ramírez et al. 2015). When growing on olive mill wastes as a carbon source, the surfactin production reached around 0.068 g/g by *Bacillus subtilis* DSM 3256, with a surface tension value of around 30 mN/m (Maass et al. 2016). The use of olive cake in solid-state fermentation (SSF) has also been described to produce up to 30.67 mg of crude lipopeptide biosurfactant per gram of solid material using *B. subtilis* SPB1 (Zouari et al. 2014). Gudiña et al. (Gudiña et al. 2016) have also evaluated the agro-industrial waste OMW as an inducer of RL production in a *P. aeruginosa*, a culture media was prepared to contain corn steep liquor (10%, v/v) and sugarcane molasses (10%, w/v), supplemented with OMW at concentrations between 5 and 25% (v/v) (used without any previous treatment). This claimed low-cost culture medium had allowed the production of 5.1 g/L of RLs at a tank fermenter scale. Its Rls have exhibited a very low CMC (13 mg/L) (Gudiña et al. 2016).

**Table 4:** Main studies on the biosurfactant production using winery and olive oil residues

Substrate(s)	Pretreatment	Strain	Biosurfactant type	Biosurfactant production (mg/L)	References
Winery wastes					
Trimming wine shoots	Acid hydrolysis + detoxification	*Lactobacillus pentosus* CECT-4023^T^	Bioemulsifier	6500	(Moldes et al. 2007)
Trimming wine shoots	Acid hydrolysis + detoxification	*Lactobacillus acidophilus* CECT-4179 and *Debaryomyces hansenii* Y-7426	Not characterized	NA	(Portilla et al. 2008)
Grape marcs	Acid hydrolysis	*Lactobacillus pentosus* CECT-4023^T^	Bioemulsifier	4.8	(Portilla-Rivera et al. 2008)
Trimming wine shoots + wine lees	Acid hydrolysis + enzymatic hydrolysis (shoots) Recovery of tartaric acid (lees)	*Lactococcus lactis* ssp. lactis CECT-4434	Not characterized	1.5	(Rodríguez et al. 2010)
Wine lees	No	*Lactococcus lactis* ssp. lactis CECT-4434	Glycolipopeptide	6.64	(Vera et al. 2018)
Grape marcs (nonfermented) (white)	Raw broth 80% (v/v)	*Burkholderia thailandensis* E264	Safe long-chain rhamnolipids	1070	Our study
Olive oil wastes					
Olive oil mill effluent (OOME)	Dilution + NaNO_3_	*Pseudomonas* sp. JAMM	Rhamnolipid	1400 >	(Mercadé et al. 1993)
Olive mill waste (OMW)	NO	*B. subtili* N1 and *P. aeruginosa* PAO1	Surfactin and Rhamnolipid	3.12 (Surfactin) 191.46 (Rhamnolipid)	(Moya Ramírez et al. 2015)
Olive mill waste (OMW)	i) acid hydrolysis ii) enzymatic hydrolysis iii) acid hydrolysis + enzymatic hydrolysis	*B. subtilis* N1 and *P. aeruginosa* PAO1	Surfactin and Rhamnolipid	26.5 (Surfactin) 299 (Rhamnoòipid)	(Moya Ramírez et al. 2016)
Olive mill waste (OMW)	NO	*P. aeruginosa* #112	Rhamnolipid	5100	(Gudiña et al. 2016)
Olive mill pomace (OMP)	Autoclaved in the culture medium 2% (w/v)	*Burkholderia thailandensis* E264	Safe long-chain rhamnolipids	300	Our study

Previous studies have described various forms of residues derived from agro-industrial processes that have been explored for biosurfactants’ production, e.g., oil processing wastes, starch waste, sugar industry wastes, fruit, and vegetable wastes, distillery waste, and animal fat, and others (Singh et al. 2019). However, little is currently known regarding the RL production from nonpathogenic microorganisms (Singh et al. 2019). In this work, we have paid attention to agricultural biomass resulting from the industrial production of wine and olive oil. We accordingly summarized the main studies existing in the literature on biosurfactant production from those two agricultural sectors (Table 4). Lignocellulosic materials are among the most abundant biomass sources available on earth, and they are the major constituents of various wastes from industries, forestry, agriculture, and municipalities (Makkar et al. 2011; Faria et al. 2019). Economically, lignocellulosic agriculture wastes as biomass feedstocks can be used for producing valuable biomolecules, e.g., biosurfactants (Makkar et al. 2011). Within the winery residues, few studies have reported that some forms of lignocellulosic resulting from pruning waste of wine stocks could be used as substrates for the production of biosurfactant (Table 4). Bustos et al. (2007) and Moldes et al. (2007) have reported the use of trimming vine shoots hydrolyzates as substrates for lactic acid production and another class of biosurfactants by *Lactobacilli* strains. Similarly, the hemicellulosic hydrolyzates from trimming vine shoots’ residues have been reported for the simultaneous production of biosurfactants and lactic acid by *L. acidophilus* and *Debaryomyces hansenii*, respectively (Portilla-Rivera et al. 2008) (Table 4). On hemicellulosic hydrolyzates from trimming vine shoots, its extracted biosurfactant has been described for reducing the surface tension to around 24.5 mN/m, in the presence of yeast extract and other nutrients (Portilla et al. 2008). In viticulture, a massive amount of grape marcs is produced after grape crushing during winemaking processes. Some of these grape marcs are usually distilled into wineries to recover ethanol that is used in the production of spirituous liquors (Makkar et al. 2011). However, large amounts of distilled grape marcs remain unutilized, containing hemicelluloses and organic acids that can be utilized to produce useful biomolecules such as lactic acid and biosurfactants (Makkar et al. 2011). To tackle the cost of production, Rivera et al. (2007) have evaluated the sugar-containing liquors from hydrolyzates of distilled grape marcs as media for the lactic acid and biosurfactants production (Table 4). They have reported a low yield of 4.8 mg/L of intracellular biosurfactants, equivalent to 0.60 mg of intracellular biosurfactant per gram of sugar using *L. pentosus* (Rivera et al. 2007). Another study has also shown that a *Bacillus tequilensis* strain ZSB10, isolated from Mexican brines, has been found to producing extracellular and cell-bound biosurfactants, likely lipopeptides for *Bacillus* genus, via culture broths formulated from hydrolysates obtained from cellulosic and hemicellulosic fractions taken from vine-trimming waste mixed with mineral medium (Cortés-Camargo et al. 2016). They obtained crude extracellular biosurfactant production of 1.52 g/L, an ST reduction of 38.6 mN/m, a CMC of 177 mg/L, and an EI of 47%, with kerosene (Cortés-Camargo et al. 2016) (Table 4). The lees, resulting from the last step of vinification, are another type of wastes that could be used as a nutrient in some low-cost fermentative media (Bustos et al. 2004). Given the lees, dead yeasts represent a potential source of nutrients, particularly after being subjected to hydrolysis. Due to these interesting characteristics, winery lees are used as a supplemented nutrient in media containing hydrolysates trimming vine shoots (Rodríguez et al. 2010) or whey (Vera et al. 2018) for the production of biosurfactant using *Lactococcus lactis* (Table 4). Within this context, our study has highlighted another class of biosurfactants, i.e., RLs, which are highly required for ecofriendly industrial applications such as soil washing and soil bioremediation. It has been demonstrated that a significant amount of substrate has been in NB + Glyc medium for cell growth during the log and early stationary phases, allowing a high microbial density (Fig. 2) (Victor et al. 2019). This had suggested the presence of a metabolic control system that slows metabolic RL production when carbon substrate concentration reached below a threshold level (Funston et al. 2016). Accordingly, this observation is not evident in the case of winery residues used in the present study, allowing lower cell density than that observed with a rich medium containing peptone and yeast extract. This could also be explored on mixtures of agricultural residues with various compositions as substrates using novel techniques such as metatranscriptomics to understand the natural RL pathways due to this strain’s natural first habitat, which is a rice field soil (Dubeau et al. 2009).

Regarding the RLs’ properties, it was shown previously that the strain E264 was able to lower the surface tension to around 43 mN/m with a CMC of 250 mg/L, when growing on NB + 4% Glyc (Dubeau et al. 2009). In another study, the (CMC) of the purified extract has determined to reach around 125 mg/L, while the surface tension is lowered to 43 mN/m (Elshikh et al. 2017). Possible explanations of our slightly higher CMCs values might be the presence of impurities in the crude extracts, the bacterial growth conditions, and the media compositions, and the abundance of RL congeners. To the best of our knowledge, our study studied highlighted the stability of long-chain RLs from *B. thailandensis* E264 for the first time*.* However, the variation between the surface tension and the emulsifying activity under the effect of pH was previously reported for Rha-Rha-C10-C10 synthesized by some *Pseudomonas* strains (Lovaglio et al. 2011). Lower chain RLs tend to precipitate at low pH, which explained the decrease of E24 and ST at pH 2. Moreover, the stability and emulsifying activity are raised at alkaline conditions due to electrostatic effects that occur around the micelles (Lovaglio et al. 2011). Similarly, Khademolhosseini et al. (2019) have demonstrated that *Pseudomonas*’ RLs are thermostable over a wide range of temperatures (40–121 °C) and that the high temperatures do not have significant effects on their chemical structures (Khademolhosseini et al. 2019). In contrast, depending on the hydrophobic substrates used for the emulsification capacity, we noted a variation regarding the RLs stabilities extracted from *P. aeruginosa*. For instance, on crude oil as a hydrophobic model, Chebbi et al. (2017b) have reported higher stabilities of RLs by *P. aeruginosa* strain W10 from 0 to 150 g/L, while Amani et al. (2013) have shown that the values of the ST resisted well against salt even at concentrations up to 25 g/L. Our results are in favor of less downstream processing required to generate a pure RL product from cell-free supernatant, though more efforts are needed to increase the production at an industrially relevant level. For the quantification of RL for the detection and quantification of RLs, most studies are both indirect and/or inaccurate leading to significant inaccuracies in their concentrations produced (Funston et al. 2016). Regarding the results found on olive oil residues, a low number of RLs congeners have been detected when corn oil using was used as a substrate by the same strain E264 (Kourmentza et al. 2018). We used for the first time the UPLC-MS method to detect and estimate RL congeners’ abundances on those agricultural residues allowing high levels of accuracy. This technique could be applied for comparing several RLs profiles using various carbons sources, including agricultural wastes, by taking into account the RL gene expression.

In general, our results revealed that nonfermented grape marcs and olive pomace residues, derived from the white winemaking process and olive oil extraction, respectively, could be used as economical substrates for the production of biosurfactants (e.g., long-chain RLs), which are in agreement with the concept of reevaluation of agricultural wastes to applying the CE principals. Moreover, the nonpathogenic nature of the selected organism and the purity shown by the crude RL extract suggested the possible use of these biomolecules directly in industrial practices or in soil remediation. A future perspective could be the reevaluation of various safe RL producers existing in all the bacteria collections for their capacities to produce those microbial glycolipids, and the possibility of gathering all the bacteria with a detailed big database containing the yields, the RLs congeners, the physiological properties, incubation time, other interfering metabolites, such as polyhydroxyalkanoates (PHAs). Parallelly, we would investigate the bacterial molecular mechanisms (e.g., quorum sensing) that are responsible for this valuable biomolecule synthesis. Overall, the safe RL from E264 has exhibited promising stabilities over wide ranges of temperature, pH, and salinity, similar to that produced by *P. aeruginosa*. For that, these biomolecules could also be applied in the bioremediation studies, which require performance and stability characters in contaminated soils. To replace the well-known *P. aeruginosa* as the RL production using the strain E264 demonstrated interesting patterns and characteristics, which makes this strain an excellent candidate for further study even on agricultural wastes substrates. Further comparative studies are also important using those agricultural residues at 25 °C on this promising strain. Advanced optimization methods on winery residues are needed to increase the RL yields by increasing the agricultural substrate’s degradability while better considering the molecular pathways involved by the biosurfactant producer.

## Supplementary Information

ESM 1 (PDF 1146 kb)

## Data Availability

The datasets generated during and/or analyzed during the current study are available from the corresponding author on reasonable request.
