# Supplementary material for: Burkholderia thailandensis E264 as a promising safe rhamnolipids’ producer towards a sustainable valorization of grape marcs and olive mill pomace
Source: Appl Microbiol Biotechnol. 2021 Apr 20;105(9):3825–42. doi: 10.1007/s00253-021-11292-0 (PMC8102290; doi:10.1007/s00253-021-11292-0)
Supplement: Supplementary file 1 — (PDF 1146 kb) [file 253_2021_11292_MOESM1_ESM.pdf]

SUPPLEMENTARY MATERIAL

APPLIED MICROBIOLOGY AND BIOTECHNOLOGY

Alif Chebbi[1] • Massimiliano Tazzari[1] • Cristiana Rizzi[1] • Franco Hernan Gomez Tovar[2], Sara Villa[1]• Silvia Sbaffoni[3]• Mentore Vaccari[2] • Andrea Franzetti[1]🖂

[1] Dept. of Earth and Environmental Sciences -DISAT, University of Milano-Bicocca, Piazza della Scienza 1 - 20126 Milano, Italy

[2] Dep. of Civil, Environmental, Architectural Engineering, and Mathematics, University of Brescia, Via Branze 43, 25123 Brescia, Italy

[3] Sustainability Department, Resource Valorisation Lab, Casaccia Research Center, ENEA, Via Anguillarese 301, 00123 Rome, Italy

🖂 Prof. Andrea Franzetti,

Dept. of Earth and Environmental Sciences -DISAT, University of Milano-Bicocca, Prof. Andrea Franzetti - andrea.franzetti@unimib.it

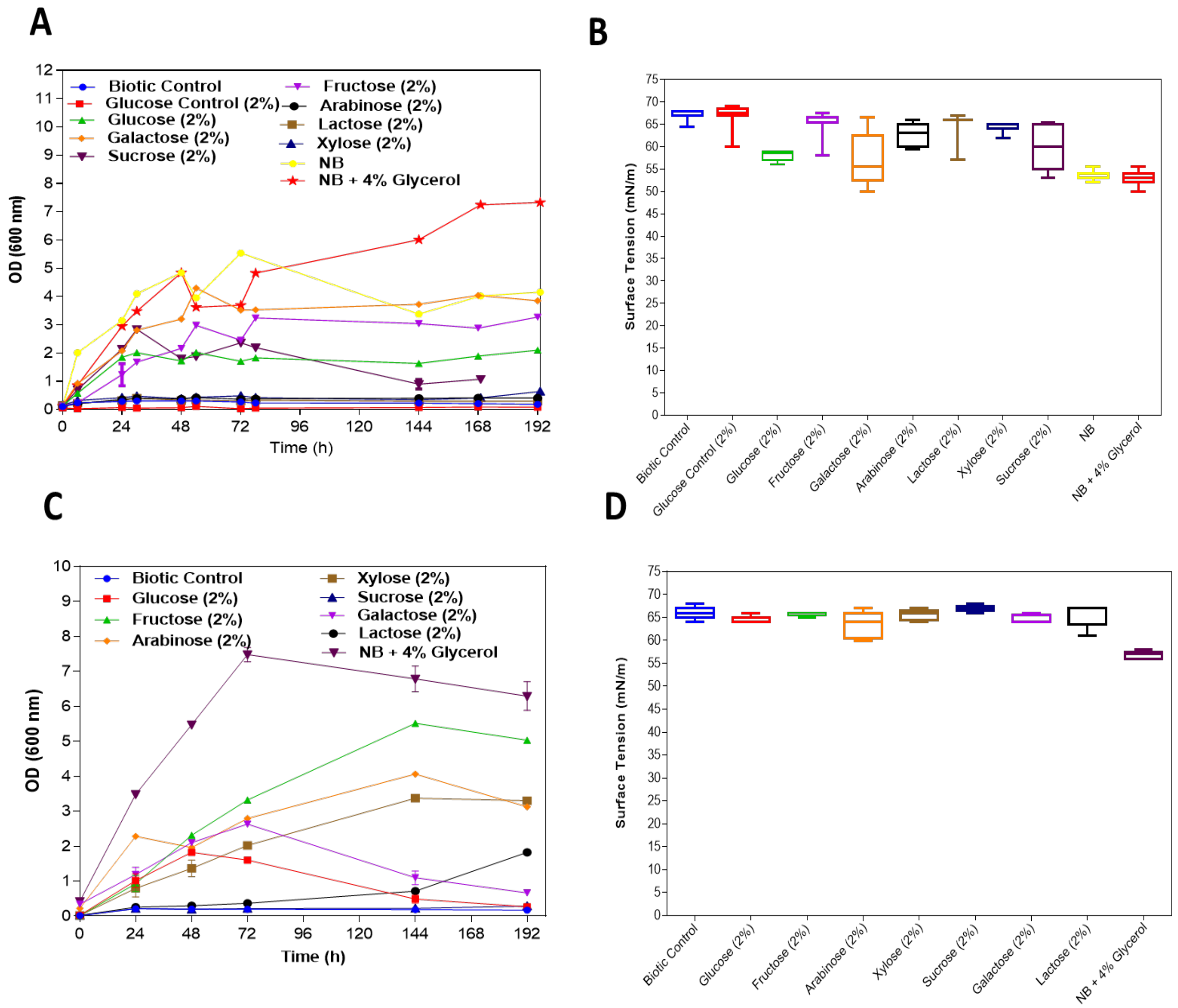

Figure S1. Monitoring the growth (OD 600 nm) and the corresponding boxplot of surface tension reductions (mN/m) on various substrates by *P. chlororaphis* DSM 50083 (**A, B**) and *P. kururiensis* DSM 13646 (**C, D**) (for sugars in MSM medium) at 30 °C and 150 rpm

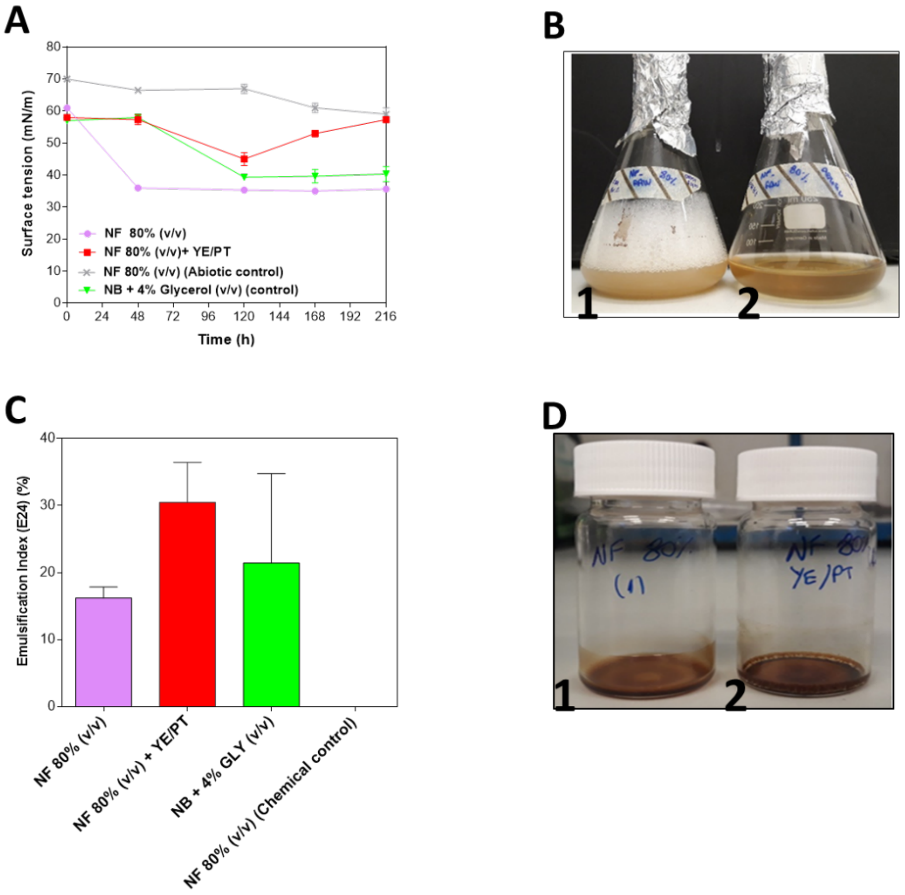

Figure S2. (**A**) Monitoring the surface tension reduction (mN/m) of *B. thailandensis* E264, in the presence of nonfermented grape marcs (NF) and fermented grape marcs (F) fractions, in MSM medium during 240 h at 30 °C and 150 rpm. Cultures on non fermented grae marcs (1) and chemical control (2) (**B**). Emulsification potential of cell-free supernatant on NF fermented grape marcs 80% (v/v), 80% (v/v) + YE/PT, and NB +4% Glycerol (Glyc) (v/v) and chemical control (NB 80%) (**C**). Extracted crude rhamnolipids from NF fermented grape marcs 80% (v/v), 80% (v/v) + YE/PT (volume culture around 100 ml) (**D**). YE: Yeast extract (0.3%, w/v), PE: Peptone (1.5%, w/v)

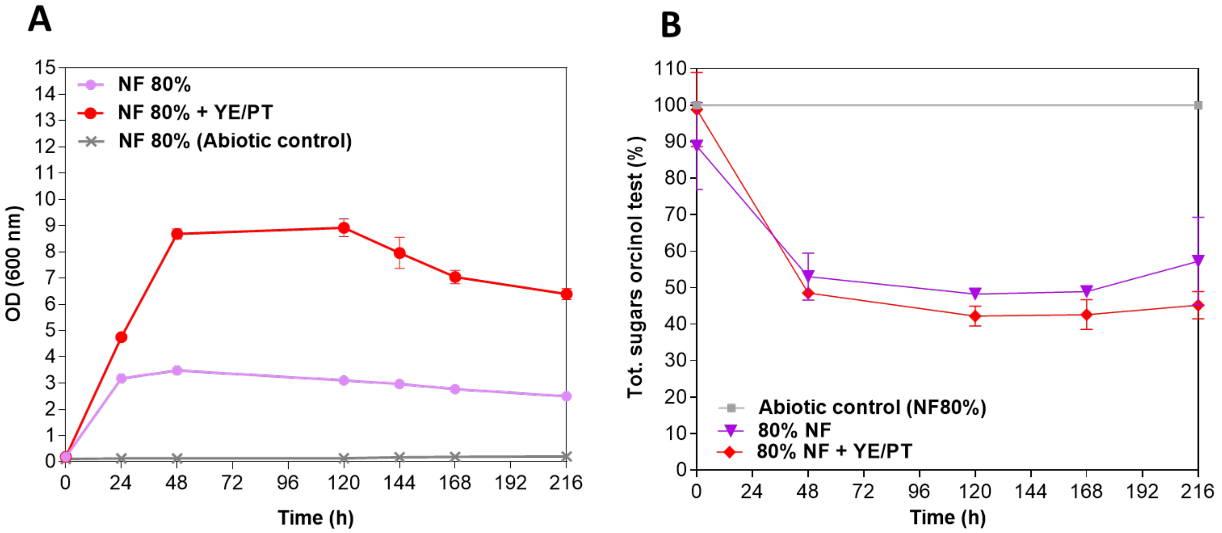

Figure S3. Monitoring the growth (OD 600 nm) (**A**) and the degradation of sugars in MSM medium supplemented with the soluble fraction of nonfermented grape marcs (NF) at (80 %; v/v) by *B. thailandensis* E264 at 30 °C and 150 rpm using orcinol test. n=4; YE: Yeast extract (0.3%, w/v) , PE: Peptone (1.5%, w/v)

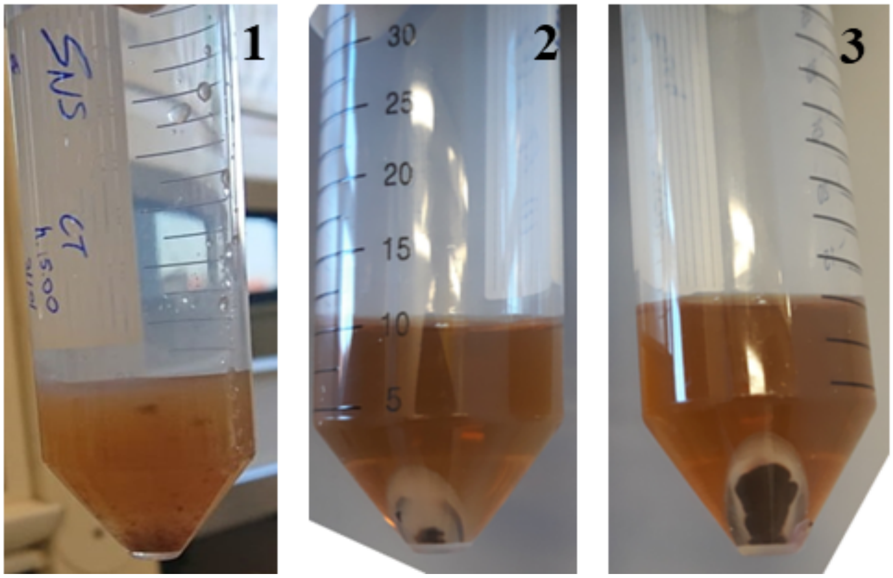

Figure S4. The difference in E264 biomass pellets after centrifuging 10 ml of culture at 8000 rpm for 15 min for the abiotic control (1) and E264 on olive mill pomace (OMP) at 2% (w/v) (2) and at 5% (w/v) (3)
